# Physicochemical Stability and Sensory Properties of Salad Dressing Formulated With Fish Oil‐in‐Water Pickering Emulsions

**DOI:** 10.1002/mnfr.70612

**Published:** 2026-10-12

**Authors:** Thang Tran, Grethe Hyldig, Betül Yesiltas, Ana C. Mendes, Ioannis S. Chronakis, Charlotte Jacobsen

**Affiliations:** ^1^ National Food Institute Technical University of Denmark Kongens Lyngby Denmark

**Keywords:** oxidative stability, physical stability, Pickering emulsions, salad dressings, sensory

## Abstract

Pickering emulsions stabilized with food‐grade particles have gained attention for food applications to deliver bioactive compounds. Fish oil, a rich source of omega‐3 PUFAs, offers numerous health benefits, but it is highly prone to lipid oxidation. While fish oil Pickering emulsions have shown potential in enhancing oxidative stability, their application in real food systems has not been explored extensively. This study investigated the physicochemical stability and sensory properties of salad dressings fortified with fish oil Pickering emulsions in comparison with other dressings fortified with neat fish oil and conventional emulsions. The dressings were stored for 4 weeks at room temperature in the dark. Analysis of droplet size indicated structural stability of Pickering emulsions in the dressings. Analysis of peroxide value and tocopherols showed that salad dressings fortified with fish oil Pickering emulsions exhibited the slowest formation of lipid hydroperoxides. In addition, the formation of volatile compounds also emphasized the effects of fish oil Pickering emulsions. Sensory evaluation revealed no significant physical deterioration across samples, and salad dressings fortified with Pickering emulsions received the lowest scores for rancid‐associated odors. These findings highlight the potential of Pickering emulsions to enhance both the oxidative and sensory stability of omega‐3 delivery systems when incorporated in salad dressing.

## Graphical abstract

Salad dressings fortified with omega 3 rich fish oil Pickering emulsions can maintain physical stability while enhancing oxidative and sensory stability. Fish oil Pickering emulsions were stabilized with protein based Janus particles from zein and sodium caseinate via an electrohydrodynamic co jetting technique. This finding highlights the potential of Pickering emulsions for omega 3 delivery systems.

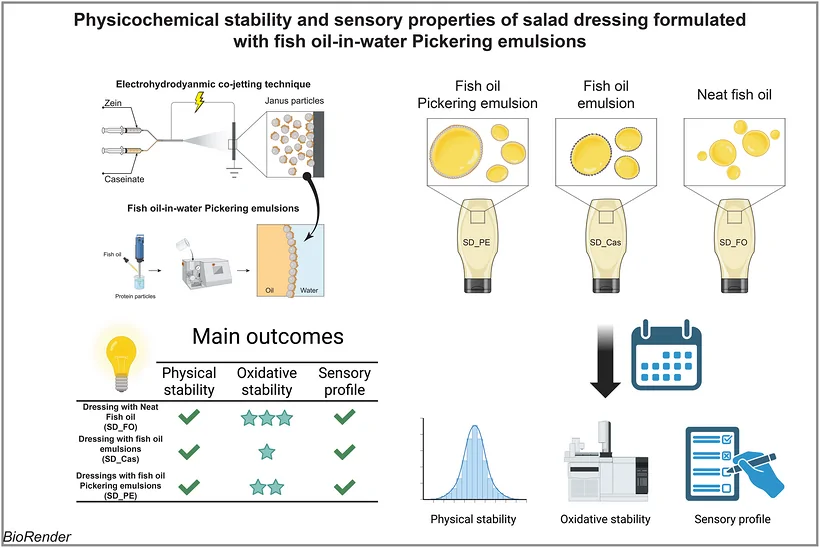

**Abbreviations**
AOCS: American Oil Chemists’ Society
min: minute(s)
PV: Peroxide value
SD_Cas: salad dressings fortified with fish oil‐in‐water emulsions stabilized with sodium caseinate
SD_FO: salad dressings fortified with neat fish oil
SD_PE: salad dressings fortified with fish oil‐in‐water Pickering emulsions stabilized with Janus particles

## 1 Introduction

Food Pickering emulsions, which are emulsions stabilized with food‐grade particles, have potential applications in delivering hydrophobic bioactive compounds [1], and such emulsions have been incorporated into various food products [2, 3, 4]. However, their use as a delivery system for fortifying foods with omega‐3 oils has not been extensively explored. This may be partly due to challenges associated with the high susceptibility of omega‐3 oils to lipid oxidation [5]. Therefore, evaluating the potential of food Pickering emulsions as lipid‐delivery systems for incorporating omega‐3 oils into diverse food products appears essential to support the dietary intake of omega‐3 polyunsaturated fatty acids (PUFAs).

Distinct advantages in delivering hydrophobic bioactive compounds arise from the natural origin of the Pickering particles and their effective stabilizing mechanisms. Natural Pickering particles, fabricated from food‐grade ingredients or bio‐based materials, have exhibited great potential for food applications in plant‐based foods or as an alternative to animal‐derived food ingredients. For example, plant‐based Pickering particles, such as cellulose nanofibrils [6] and gliadin colloidal particles [7], have been used to produce Pickering emulsions. These emulsions are potential alternatives to animal‐derived food ingredients, and they are advantageous due to their natural origin and compatibility with clean‐label formulations. In addition, lime residue‐derived cellulose nanofibers were used to stabilize soybean oil Pickering emulsions, enabling a reduction in egg yolk content from 4% to 2% when incorporated into salad dressings [8]. Pickering particles can enhance the textural and rheological properties of food systems based on their effective mechanisms of Pickering stabilization. An example of this is the ability of gliadin colloidal particles and shea butter to enhance foam stability in non‐dairy whipping cream [9], or gel strength in surimi [4]. Despite these promising functionalities, research on the application of Pickering emulsions in food systems remains limited, particularly regarding their impact on oxidative stability and sensory properties in food products fortified with PUFAs‐rich oils such as fish oil. Oils rich in omega‐3 PUFAs, including fish oil, have been incorporated into various food products to promote the intake of omega‐3 PUFAs, which is widely associated with health benefits such as reduced inflammation, improved cardiovascular health, and enhanced cognitive function [10].

However, fish oil is highly prone to lipid oxidation, which leads to undesirable sensory changes and poses challenges for food fortification [11, 12]. Lipid oxidation takes place through a chain reaction, comprising three main steps: initiation, propagation, and termination [13, 14]. In the reaction chains of lipid oxidation, lipid hydroperoxides are primary oxidation products that can be broken down to volatile oxidation products. The volatile compounds that are the secondary products of lipid oxidation can cause undesirable sensory changes, like gluey, green, or rancid off‐odors. To counteract the challenges associated with lipid oxidation, emulsion‐based delivery systems have been evaluated to deliver omega‐3 oils into food products, particularly emulsified foods, such as mayonnaise and salad dressing [12].

Successfully fortifying salad dressings with fish oil could therefore provide a practical and appealing way to increase omega‐3 intake. Previous studies have explored various strategies to incorporate fish oil into salad dressings, aiming to improve oxidative stability and maintain sensory properties; these include the use of antioxidants [15], conventional emulsions [16], and encapsulated fish oil produced by spray‐drying or electrospraying [17]. Microencapsulation, for example, involves enclosing fish oil within a protective matrix to shield it from lipid oxidation.

Despite numerous approaches, to the best of our knowledge, no studies have investigated the direct incorporation of fish oil Pickering emulsions into salad dressings, leaving the potential of this approach unexplored. A recent study demonstrated that fish oil Pickering emulsions stabilized with zein–caseinate Janus particles exhibited excellent oxidative stability by effectively limiting the formation of secondary volatile compounds such as 2,4‐heptadienal and hexanal [18]. These zein‐caseinate Janus particles, produced by electrohydrodynamic co‐jetting, exhibited particle size (around 0.6 µm) and interfacial activity suitable for Pickering stabilization. In addition, due to the combination of sodium caseinate, which has antioxidant properties [19], and zein, the resulting zein‐caseinate Janus particles provided a dual protective mechanism against physical and chemical destabilization [18]. Therefore, the potential of using this Pickering emulsion for fortifying salad dressings with fish oil warrants further investigation.

The aim of this study was to investigate the effects of different fish oil fortification approaches on the physical stability, oxidative stability, and sensory profile of salad dressings, which were used as a model food system representing widely consumed emulsified products. The fortification approaches included neat fish oil, conventional emulsions, and Pickering emulsions. Fish oil‐in‐water emulsions were prepared using sodium caseinate or zein‐caseinate Janus particles to produce conventional emulsions and Pickering emulsions, respectively. The physicochemical stability and sensory properties of salad dressings were evaluated during a 4‐week storage period.

## 2 Materials and Methods

### 2.1 Materials

In all cases, the main ingredients used to prepare salad dressings (350 g) included cod liver fish oil (2.5 wt%, Vesteraalens A/S, Sortland, Norway), rapeseed oil (22.5 wt%), potassium sorbate (0.1 wt%), Grindsted FF5128 stabilizer (1.2 wt%), vinegar (6.0 wt%), whey protein concentrate (0.08 wt%), and ultrapure water. The fatty acid profile of the cod liver fish oil, reported in previous studies [20, 21], included C14:0 (3.7% of total fatty acids); C16:0 (9.0%); C16:1n‐7 (9.6%); C18:0 (1.9%); C18:1n‐9 (15.8%); C18:1n‐7 (4.5%); C18:2n‐6 (2.4%); C18:3n‐3 (1.0%); C20:1n‐9 (13.7%); C20:5n‐3 (8.4%); C22:1n‐11 (5.6%); and C22:6n‐3 (10.7%). Rapeseed oil, vinegar, and whey protein concentrate were purchased from a local supermarket and used without further treatment.

Sodium caseinate powder (MIPRODAN 30, Arla Foods Ingredients, Viby, Denmark) and zein (Sigma–Aldrich, St. Louis, MO, USA) were used to prepare fish oil emulsions.

### 2.2 Salad Dressings Fortified With Fish Oil

Salad dressings with 2.5 wt% fish oil were prepared by following previous publications with slight modifications [15, 16, 17]. To prepare salad dressing with neat fish oil, a Stephan Universal Mixer (Stephan, Hameln, Germany) was used, operating at 1200 rpm under vacuum (‐40% on the vacuum gauge), with circulating water for cooling. Potassium sorbate and whey protein concentrate were first dissolved in ultrapure water and added into the mixer. Separately, Grindsted FF5128 powder was blended with a portion of the rapeseed oil and added to the mixer. These ingredients were mixed for 2 min under the same operating conditions.

Subsequently, mixing was continued for an additional 3 min without vacuum, during which the remaining rapeseed oil and vinegar were gradually added, followed by the slow addition of fish oil. The mixture was then mixed for another 2 min under vacuum at the same operating conditions. The salad dressing fortified with neat fish oil (SD_FO) was used for the storage experiments.

### 2.3 Salad Dressings Fortified With Fish Oil‐in‐Water Emulsion

Salad dressings fortified with fish oil‐in‐water emulsions (conventional or Pickering emulsions) were prepared by first producing the emulsions and then incorporating them into the dressing during mixing, immediately after emulsification.

#### 2.3.1 Salad Dressing Fortified With Conventional Emulsion

A conventional emulsion was prepared using fish oil and a sodium caseinate solution. Sodium caseinate powder was dissolved in acetate buffer (10 mM, pH 3) to obtain a sodium caseinate solution (0.2 wt%). This solution was emulsified with fish oil to produce a conventional emulsion (5 wt% fish oil and 0.2 wt% caseinate) using two‐step emulsification process. First, fish oil was homogenized with the sodium caseinate solution at 16000 rpm for 3 min using a Ystral homogenizer (X10/25, Ystral GmbH, Ballrechten‐Dottingen, Germany) to produce a coarse emulsion. In the second step, the coarse emulsion was homogenized  using a high‐pressure homogenizer (Microfluidizer, M110L Microfluidics, Newton, MA, USA) equipped with a ceramic interaction chamber (CIXC, F20Y, 75 µm internal diameter) at 62 MPa for three passes.,

Salad dressings were fortified with the conventional emulsion using a process similar to that used to prepare SD_FO, with the only difference being that the fish oil and part of the water were replaced with the prepared conventional emulsion. Specifically, the conventional emulsion was gradually added after the incorporation of rapeseed oil and vinegar during salad dressing preparation. The total amount of water was adjusted accordingly to ensure consistent overall composition across all samples. The salad dressing fortified with the conventional emulsion (SD_Cas) was used for the storage experiments.

#### 2.3.2 Salad Dressing Fortified With Pickering Emulsions

The Pickering emulsion was prepared using fish oil and zein‐caseinate Janus particles produced by electrohydrodynamic co‐jetting. A suspension of zein‐caseinate Janus particles (0.2 wt%) was prepared as described in our previous work [18]. Fish oil‐in‐water Pickering emulsions stabilized with zein‐caseinate Janus particles (5 wt% fish oil and 0.2 wt% particles) were prepared using the same two‐step emulsification process as that used to prepare the conventional emulsion stabilized with sodium caseinate.

Thereafter, the resulting fish oil Pickering emulsion was slowly added during salad dressing preparation as described above. The salad dressing fortified with the fish oil Pickering emulsion (SD_PE) was used for the storage experiments.

### 2.4 Storage Experiment of Fish Oil‐Fortified Salad Dressing

All salad dressings were prepared in duplicate, divided into five bottles, and stored in the dark at room temperature (20°C) for 4 weeks. One bottle from each batch was sampled weekly (from week 0 to week 4) to evaluate physicochemical properties. This setup ensured that lipid oxidation progressed in a comparable manner across all samples throughout the storage experiment. Sensory evaluation was performed on samples collected at weeks 0, 2, and 4.

Droplet size analysis and sensory analysis were performed on the same day that samples were collected. For analysis of lipid oxidation, salad dressing samples were purged with *N*_2_ and stored at −40°C until analysis.

### 2.5 Characterization of Physical and Chemical Properties of Salad Dressings

#### 2.5.1 Analysis of Oil Droplet Size Distribution and Morphology

Dressing samples (1 g) were re‐suspended in 1% SDS solution (5 g) and sonicated for 15 min prior to droplet size analysis (Mastersizer 3000, Malvern Instruments Ltd., Worcestershire, UK) for size distribution.Particle sizes, volume‐weighted mean diameter D[4,3] ($D\left[4,3\right]=\frac{\sum n_{i}D_{i}^{4}}{\sum n_{i}D_{i}^{3}}\;$), and surface‐weighted mean diameter D[3,2] ($D\left[3,2\right]=\frac{\sum n_{i}D_{i}^{3}}{\sum n_{i}D_{i}^{2}}\;$) were determined, where $n_{i}$ is number of particles and $D_{i}$ is diameter of the *i* ^th^ particle. Microscopic images of oil droplets were acquired using a Primo Star microscope (Carl Zeiss, Oberkochen, Germany) at ×100 magnification with oil immersion.

#### 2.5.2 Lipid Extraction, Analysis of peroxide value and tocopherol content

Lipids were extracted from salad dressings using the Bligh and Dyer method with slight modifications [22]. The dressing samples (5 g) were mixed with 10 mL deionized water and homogenized with methanol:chloroform mixture (ratio 1:1, v/v; 30 mL) at 10000 rpm for 20 s. The mixture was centrifuged at 1402 ×*g* for 10 min at 18°C (SIGMA 4–16KS, Sigma Laborzentrifugen GmbH, Germany). The bottom layer, containing lipid extract, was collected, filtered, and used for further analysis of peroxide value (PV) and tocopherol contents.

The PV of dressings was determined using the ferric‐thiocyanate‐based spectrophotometric method (500 nm) [23], as described in previous publication [18]. PV was expressed as meq peroxide/kg oil (meq O_2_/kg oil). PV analysis was performed in duplicate.

The lipid extracts were also analyzed for tocopherol contentusing HPLC according to the American Oil Chemists’ Society (AOCS) official method (1998) [24], as described in the previous publication [18]. Standard tocopherol solutions of known concentrations were used for calculation, and the tocopherol content in the samples was determined by integrating chromatogram peak areas. Tocopherol content was expressed as µg tocopherol/g dressing. Tocopherol analysis was performed in duplicate.

#### 2.5.3 Analysis of Secondary Lipid Oxidation Products

Salad dressings were analyzed for the secondary lipid oxidation products, as previously described with slight modifications [17]. Dressings (4 g) were mixed with 30 mg of internal standard solution (4‐methyl‐1‐pentanol, 30 µg/g water). Volatile compounds from the dressing samples were released by heating the sample in a pear‐shaped flask in a water bath (45°C, 30 min) while purging with a flow of *N*_2_ at a flowrate of 150 mL/min. Once the volatile compounds were released from the sample, they passed through potassium hydroxide powder packed in S‐shaped glass tube before they were adsorbed in Tenax GR tubes [25]. These Tenax GR tubes were flushed with N_2_ (50 mL/min) for another 20 min to remove any water collected in the tubes.

Volatile compounds trapped in Tenax GR tubes were released by thermal desorption using an Automatic Thermal Desorption system (TurboMatrix 650 ATD, PerkinElmer, Norwalk, CT, USA) and analyzed with Agilent GC–MS 6890N (Agilent, Palo Alto, CA, USA), as described in previous publications [18, 26].

Volatile compounds were identified with MS spectral library (Wiley 138 K, John Wiley and Sons, Hewlett Packard), and the volatile compounds was quantified using calibration curves of standard solutions, including 1‐penten‐3‐ol, 1‐penten‐3‐one, hexanal, 2‐ethylfuran, (*E,E*)‐2,4‐heptadienal, and nonanal. A series of standard solutions with concentrations ranging from 0 to 100 µg/g was prepared. Standard solution at each concentration (30 mg) was added to a dressing sample (SD_Cas) and analyzed for volatile compounds as described above. Volatile compound analysis was performed in triplicate.

### 2.6 Evaluation of Sensory Attributes of Salad Dressings

Sensory evaluation was performed by a trained sensory panel at National Food Institute, Technical University of Denmark. The panel was specifically trained in objective descriptive analysis and the sensory evaluations were performed in a sensory lab under normal daylight and at ambient temperature according to the standards and guidelines for the design and construction of sensory lab (ISO 13300–25492 (2006); ISO 3972 (2011) & ISO 3972 (2011)/Cor 1 (2012); ISO 8586 (2012); ISO 8589 (2007); NMKL Procedure No. 6, 2023) [27, 28, 29, 30, 31, 32].

The first training sessions were used to develop a vocabulary to describe the sensory characteristics of the samples. The next three sessions were used to train the sensory panel in assessing and scoring the intensity of the selected sensory attributes. For the training, a set of samples with different sensory attributes and intensity levels were used to ensure that all experimental conditions were represented. The scale was an unstructured 15‐cm line scale with anchor points at 1.5 and 13.5 cm. The samples were poured in plastic cups with lids and stored at 20°C for one hour before serving. All samples were served in replicates and in random order and were identified by three‐digit codes.

The final vocabulary list includes Sourish, Vinegar, Sweet, Green/Hay, Oil, Glue, Varnish/Wet cardboard for Odors; White, Shiny, Bubbles, Lumps for Appearance; and Creamy, Viscous, Sticky, Rubber for Texture (evaluated using a spoon).

### 2.7 Statistical Analysis

Statistical analysis was performed using a two‑way ANOVA followed by Tukey's post hoc test in OriginPro (Version 2022, OriginLab Corporation, Northampton, MA, USA) to determine significant differences (p < 0.05).

## 3 Results and Discussion

### 3.1 Physical Properties of Salad Dressings

The physical stability of the salad dressings (SD_FO, SD_Cas, and SD_PE) was evaluated by monitoring changes in visual appearance and oil droplet size during storage. As shown in Figure 1, the visual appearance of the fortified salad dressings remained largely unchanged throughout the storage period. All samples maintained a semi‐liquid, highly viscous consistency.

**FIGURE 1:**
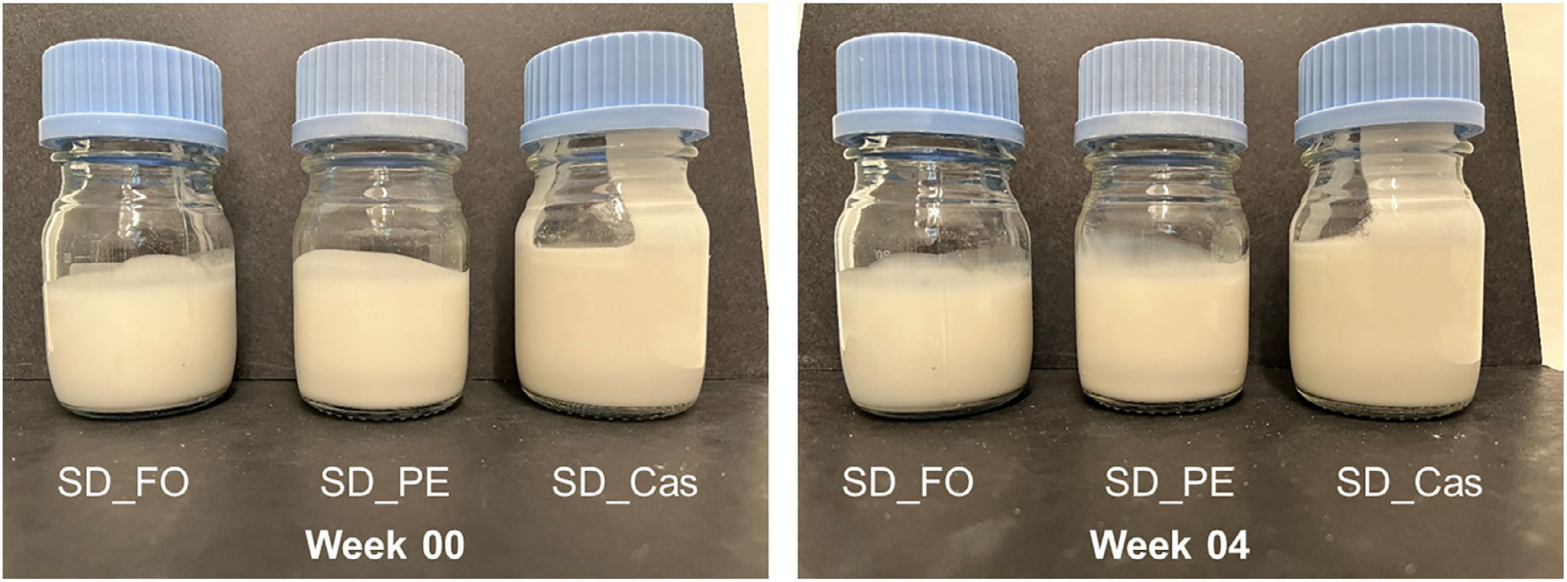
Appearance of salad dressings fortified with neat fish oil (SD_FO), fish oil‐in‐water Pickering emulsions (SD_PE), and conventional fish oil emulsions stabilized with sodium caseinate (SD_Cas) at weeks 0 and 4.

The physical stability of the salad dressings was evaluated by assessing their droplet size distribution at week 0 and week 4 (Figure 2). At week 0, right after production, the droplet size distributions were different between the fortified salad dressings. SD_FO exhibited three main populations of oil droplets at about 1.7, 13.5, and 134.9 µm, with the dominant peak observed at approximately 13.5 µm (Figure 2a). In contrast, SD_Cas showed only two main populations of oil droplets, around 8.0 µm and 92.0 µm (Figure 2b). Compared to the size distribution in SD_FO, SD_Cas showed a broader distribution of oil droplets. Two main population peaks, around 1.0 µm and 8.0 µm, were observed in SD_PE (Figure 2c). A complete absence of large oil droplets, around 100 µm, was observed in SD_PE at week 0; however, large oil droplets were observed after 4 weeks of storage (Figure 2f). It is noteworthy that the formation of a new shoulder at 1.0 µm was observed exclusively in the Pickering emulsion salad dressing (SD_PE) (Figure 2c). This feature may be related to the addition of Pickering emulsions, as a previous study with fish oil Pickering emulsions stabilized with zein‐caseinate Janus particles reported droplet sizes ranging from 0.1 to 2.0 µm [18]. It is in agreement with the research of salad dressings fortified with fish oil capsules [17]. In the study, fish oil capsules exhibited a droplet size distribution peaking at 0.3 µm, and salad dressings fortified with these capsules showed a comparable shoulder with peaks centered around 0.3–0.4 µm. In this study, the presence of the shoulder at 1.0 µm indicates that the Pickering emulsions remained structurally intact during the salad dressing production process, which can be attributed to the stability provided by protein‐based Janus particles used as stabilizers. The peak observed at 1.0 µm reflects the presence of Pickering emulsions, while the distinct peak at 8.0 µm is attributed to rapeseed oil droplets distributed throughout the food matrix, as shown in microscopic image (Figure 2i). One surprising result was the size distribution of SD_Cas (Figure 2b). Despite being fortified with fish oil emulsions stabilized by sodium caseinate, SD_Cas did not exhibit a noticeable proportion of small oil droplets. Moreover, its droplet size distribution remained consistent between week 0 and week 4 (Figure 2b,e), suggesting limited structural changes during storage. Interestingly, the incorporation of emulsions into salad dressing shifted the dominant oil droplet size distribution from 13.5 µm (SD_FO) to 8.0 µm (SD_Cas and SD_PE). This may be attributed to the non‐adsorbed proteins in the aqueous phase of fish oil emulsions. These proteins may contribute to stabilizing rapeseed oil droplets, resulting in a shift toward smaller oil droplets when either conventional or Pickering emulsions were incorporated.

**FIGURE 2:**
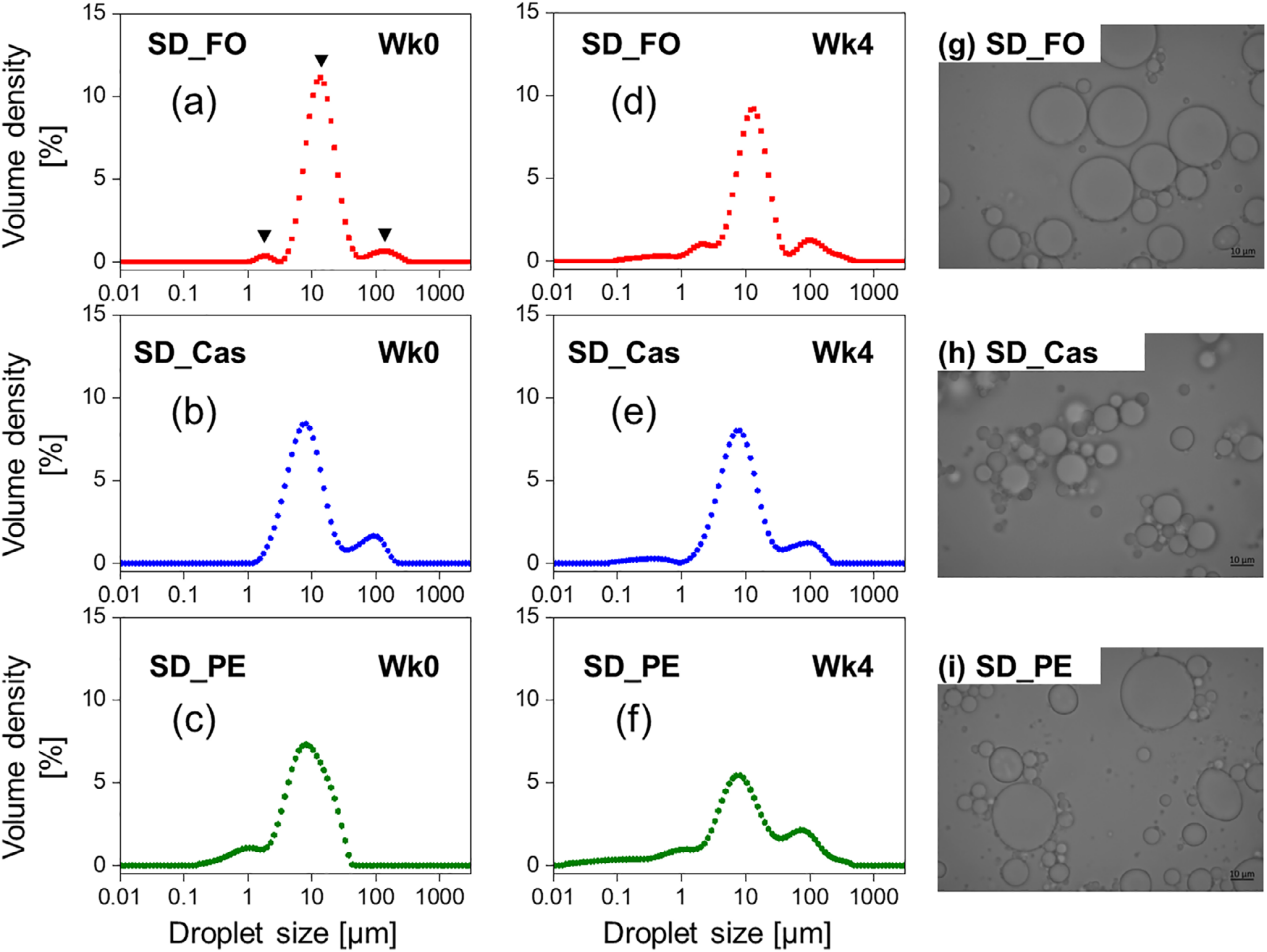
Droplet size distribution of salad dressings fortified with fish oil at weeks 0 and 4 (a—f) and representative microscopic images at week 4 (g–i).

As shown in Table 1, only a few significant differences were observed in D[4,3] and D[3,2] among salad dressings throughout the storage period (Table 1). While D[4,3] values reflect the evolution of large oil droplets, D[3,2] values in SD_PE samples suggest the presence of small droplets associated with Janus‐based Pickering emulsions during storage experiments.

**TABLE 1:** Droplet size (D[4,3] and D[3,2]) of salad dressings during storage experiments.

D[4,3], µm	Week 0	Week 1	Week 2	Week 3	Week 4	Average
SD_FO	16.59 ± 4.93^ab^	23.58 ± 1.80^ab^	23.55 ± 0.57^ab^	21.84 ± 0.48^ab^	24.69 ± 1.61^b^	22.05 ± 3.55^c^
SD_Cas	20.18 ± 0.74^ab^	18.21 ± 0.90^ab^	20.94 ± 3.09^ab^	18.26 ± 0.02^ab^	19.90 ± 0.88^ab^	19.50 ± 1.61^d^
SD_PE	18.52 ± 4.31^ab^	15.61 ± 0.02^a^	17.56 ± 0.37^ab^	16.50 ± 0.49^ab^	22.21 ± 2.07^ab^	17.96 ± 2.88^d^

D[3,2], µm	Week 0	Week 1	Week 2	Week 3	Week 4	Average
SD_FO	9.13 ± 4.13^a^	6.77 ± 0.90^ab^	8.40 ± 3.10^ab^	5.36 ± 4.92^ab^	5.86 ± 0.91^ab^	7.10 ± 2.86^c^
SD_Cas	7.48 ± 0.53^ab^	3.77 ± 0.42^ab^	4.71 ± 0.69^ab^	4.66 ± 0.32^ab^	4.17 ± 0.97^ab^	4.96 ± 1.46^c^
SD_PE	1.78 ± 0.82^ab^	0.85 ± 0.16^b^	0.77 ± 0.21^b^	0.76 ± 0.02^b^	0.75 ± 0.11^b^	1.48 ± 1.49^d^

*Note*: Superscript letters (a and b) indicate significant differences in means between types of salad dressings across storage weeks. Superscript letters (c and d) in the “Average” column indicate significant differences among types of dressings. Means that do not share a letter are significantly different.

The D[4,3] values of SD_FO fluctuated from 16.59 ± 4.93 to 24.69 ± 1.61 µm between week 0 and week 4, with an average D[4,3] value around 22.05 ± 3.55 µm over the storage period. SD_PE also exhibited a fluctuation in D[4,3] values, ranging from 15.61 ± 0.02 to 22.21 ± 2.07 µm, with an average D[4,3] value around 17.96 ± 2.88 µm during this period. Similarly, the average D[4,3] value for SD_Cas was 19.50 ± 1.61 µm over the 4‐week storage period. A significant difference was observed between the D[4,3] values of SD_FO and the other two dressings, with SD_FO having a notably larger average droplet size (22.05 ± 3.55 µm) compared to SD_PE (17.96 ± 2.88 µm) and SD_Cas (19.50 ± 1.61 µm). This aligns well with the previously noted shift in dominant droplet size from 13.50 µm (SD_FO) to 8.00 µm (SD_Cas and SD_PE), as mentioned above. The increase in D[4,3] values from week 0 to week 4, as shown in Table 1, indicates that oil droplet coalescence likely occurred during storage. In contrast, a previous study showed significant differences in D[4,3] values at week 0 for salad dressings fortified with fish oil microcapsules [17]. However, in the present study, no significant differences in D[4,3] values among the dressings were observed at  week 0.

The D[3,2] values of dressings are also summarized in Table 1. For SD_PE, D[3,2] values ranged from 4.37 ± 0.82 to 0.75 ± 0.11 µm over the four‐week storage period, with an average value of 1.48 ± 1.49 µm. This was significantly lower than the average D[3,2] values observed in SD_Cas (4.96 ± 1.46 µm) and SD_FO (7.10 ± 2.86 µm). An unexpectedly high D[3,2] value of 4.37 µm was observed at week 0 for SD_PE. The reason for this high value remains unclear; however, this could be due to insufficient settling time for the oil droplets before the initial measurement, which could have affected the accuracy of the early droplet size readings. From week 1 to week 4, small oil droplets with diameters ranging from 0.75 ± 0.11 to 0.85 ± 0.16 µm were consistently observed, which is in agreement with previous research on fish oil Pickering emulsions stabilized by zein‐caseinate Janus particles [18]. This indicates the presence of fish oil Pickering emulsions in SD_PE, and their high physical stability after fortification during the storage experiments. In earlier experiments of salad dressings fortified with emulsion‐based delivery systems generally showed smaller droplet sizes than those containing non‐emulsion encapsulation systems [17].

### 3.2 Oxidative Stability of Salad Dressings

#### 3.2.1 Formation of Lipid Hydroperoxides and Consumption of Tocopherols

The oxidative stability of salad dressings was evaluated by monitoring the formation of lipid hydroperoxides (Figure 3a) and the depletion of α‐tocopherol (Figure 3b). Overall, a slight increase in PV was observed in all salad dressings, while α‐tocopherol levels remained stable throughout the 4‐week storage period. Among the samples, SD_PE exhibited the slowest rate of lipid hydroperoxide formation compared to the other two dressings (SD_Cas and SD_FO), indicating enhanced oxidative stability (Figure 3a).

**FIGURE 3:**
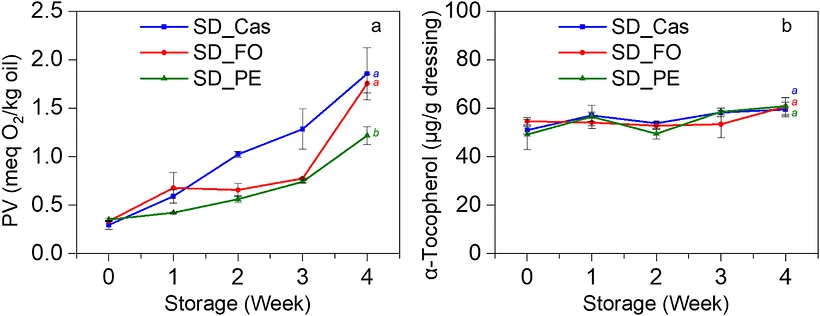
PV (a) and α‐tocopherol content (b) of salad dressings during storage experiments. Means that do not share the same subscript letter are significantly different (*p* < 0.05).

The PV of pure fish oil (0.18 ± 0.02 meq O_2_/kg oil) was lower than the PVs observed in all salad dressing samples at week 0 (0.29–0.35 meq O_2_/kg oil). This suggests that processing stages may contribute to initial lipid oxidation, as previously indicated in the literature [33]. The PV of salad dressing fortified with Pickering emulsions (SD_PE) increased from 0.35 ± 0.02 to 0.74  ±  0.01 meq O_2_/kg oil between week 0 and week 1, although this change was not statistically significant. By week 4, the PV rose gradually to 1.22  ±  0.09 meq O_2_/kg oil. Despite this increase, SD_PE maintained a lower PV than SD_Cas and SD_FO at week 4, which reached 1.86  ±  0.27 and 1.76  ±  0.10 meq O_2_/kg oil, respectively. In SD_Cas, the PV rose from 0.29  ±  0.05 to 0.59  ±  0.06 meq O_2_/kg oil in the first week, although this increase was not statistically significant, but then significantly increased to 1.02  ±  0.03 meq O_2_/kg oil at week 2 and continued rising to 1.86  ±  0.27 meq O_2_/kg oil by week 4. In contrast, SD_FO showed a gradual increase in PV from 0.33  ±  0.00 to 0.77  ±  0.01 meq O_2_/kg oil over the first three weeks, followed by a significant jump to 1.76  ±  0.10 meq O_2_/kg oil at week 4. A lag phase in PV development was observed in both SD_PE and SD_FO from week 0 to week 3, with significant increases occurring only at week 4, whereas SD_Cas exhibited a shorter lag phase, with a significant rise beginning at week 2. Overall, SD_PE exhibited the slowest rate of lipid hydroperoxide formation among the three dressings, particularly evident by week 4. This finding aligns with previous studies showing that PV in dressings fortified with fish oil and fish oil emulsions remained similar up to 22 days of storage [16]. One interesting finding is that the PV of SD_FO at week 4 in this study (1.76 meq O_2_/kg oil) was notably lower than the values reported in a previous study (approximately 6 meq O_2_/kg oil) over a similar storage period (∼ 28 days) [16]. This significant reduction in PV can be directly attributed to the use of a 4‐fold lower concentration of fish oil to replace rapeseed oil in the present formulation (2.5 wt% fish oil instead of 10 wt% fish oil in the previous study). This suggests that the amount of fish oil may be a major factor influencing lipid oxidation.

Tocopherols are natural antioxidants in fish oil and rapeseed oil, and the depletion of tocopherol homologues was monitored over time (Figures 3b and S1). At week 0, the content of α‐tocopherol was 50.94 ± 1.62, 54.61 ± 1.61, and 49.09 ± 6.22 µg/g dressing in SD_Cas, SD_FO, and SD_PE dressings, respectively. From week 0 to week 4, no significant changes in α‐tocopherol content were observed in any of the salad dressings during storage, and similar trends were found for the other tocopherols (Figure S1). It was discussed in a previous study that mixing fish oil with rapeseed oil before processing may help prevent salad dressing from oxidizing because of the protective antioxidant effects of tocopherols naturally present in rapeseed oil [16]. The tocopherol analysis showed that tocopherols were sufficient to limit lipid oxidation.

Taken together, PV and tocopherol results suggest that fish oil Pickering emulsions provide enhanced oxidative stability when added to salad dressing compared to when neat fish oil or caseinate emulsions are used as omega‐3 PUFA delivery systems. Combined with the droplet size analysis, these findings are consistent with the retention of Pickering emulsions during both the fortification process and subsequent storage.

#### 3.2.2 Formation of Secondary Volatile Lipid Oxidation Products

Overall, the salad dressings in this study formed low levels of volatile compounds, which is consistent with the previous research [17]. The evolution of six volatile compounds, including 1‐penten‐3‐ol, 1‐penten‐3‐one, hexanal, 2‐ethylfuran, (*E,E*)‐2,4‐heptadienal, and nonanal, is presented in Figure 4. Analysis of volatile compounds revealed two different trends over the 4‐week storage period. Two volatile compounds, 1‐penten‐3‐ol and 2‐ethylfuran, exhibited a clear increase in concentration from week 0 to week 4 (Figures 4a and S2a), although their levels remained below 15 ng/g sample. In contrast, all other volatile compounds did not exhibit a consistent increase throughout the storage period (Figures 4b and S2b–d). A previous study on salad dressings fortified with fish oil microcapsules also reported low levels of several volatile compounds that did not increase during storage, likely because the fish oil was already oxidized before fortification [17]. Similarly, the fluctuations without a clear increase observed in this study, for example at 50 ng/g sample for nonanal (Figure 4b), suggest that these volatile compounds were primarily formed during emulsification and fortification processes. Furthermore, the PV may have remained below a certain critical threshold and therefore was insufficient to promote substaintial formation of volatile compounds [34].

**FIGURE 4:**
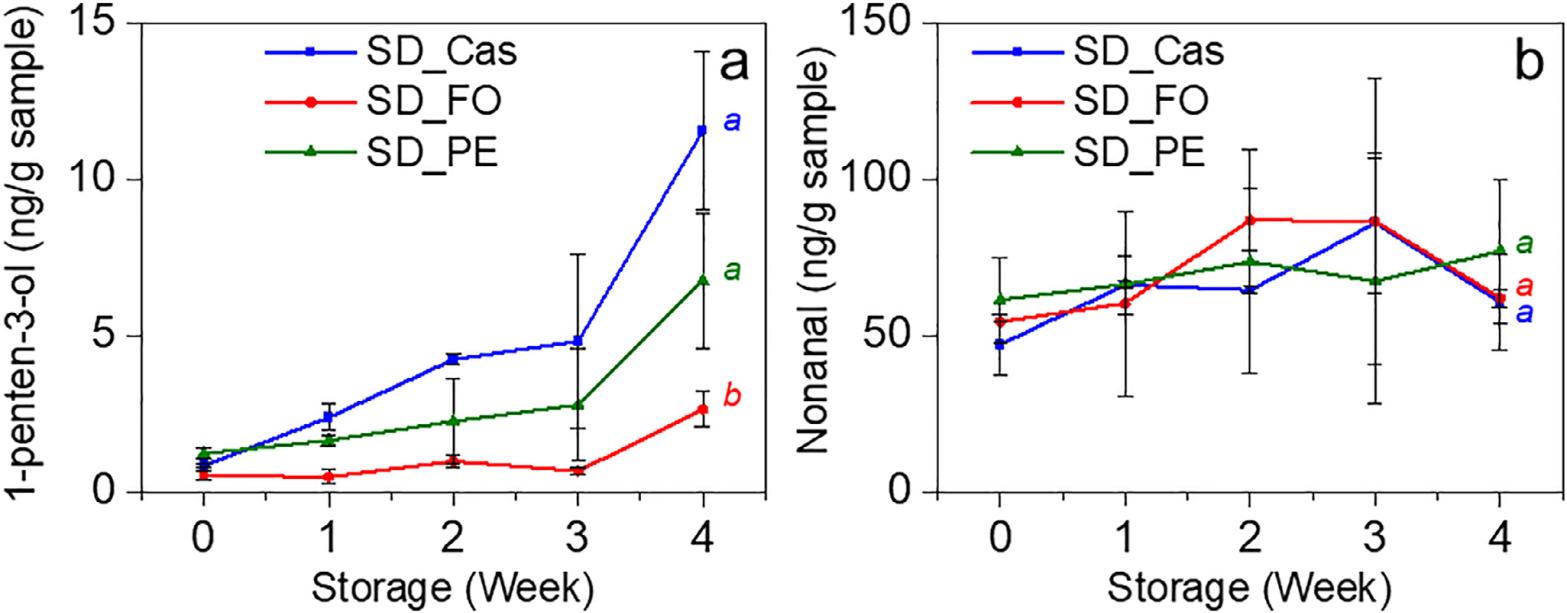
Formation of volatile compounds, (a) 1‐penten‐3‐ol and (b) nonanal, in salad dressings during storage. Means that do not share the same subscript letter are significantly different (*p* < 0.05).

As illustrated in Figure 4a, the concentration of 1‐penten‐3‐ol was similar for all three salad dressings (SD_Cas, SD_FO, and SD_PE) at the beginning of the storage experiments. In SD_FO, the concentration fluctuated between 0.52 ± 0.12 ng/g sample and 0.66 ± 0.99 ng/g sample from week 0 to week 3, then slightly increased to 2.64 ± 0.55 ng/g sample at week 4. In SD_PE, it slowly increased from 1.22 ± 0.19 ng/g sample to 2.78 ± 1.81 ng/g sample during the first three weeks and then rose to 6.75 ± 2.16 ng/g sample at week 4, which was higher than in SD_FO. In SD_Cas, the concentration of 1‐penten‐3‐ol was the highest among the three samples at week 4, reaching 11.55 ± 2.51 ng/g sample, after gradually increasing from 0.83 ± 0.07 ng/g sample at week 0 to 4.81 ± 2.78 ng/g sample at week 3. In summary, SD_FO showed the lowest concentration of 1‐penten‐3‐ol; it was followed by SD_PE, while SD_Cas showed the highest concentration of 1‐penten‐3‐ol, particularly at week 4 of the storage experiment. A similar pattern was observed for 2‐ethylfuran (Figure S2a).

The differences in the content of volatile compounds between SD_FO, SD_Cas, and SD_PE could be explained by variations in droplet size. As mentioned previously, SD_FO exhibited significantly larger droplet sizes, particularly D[4,3], compared to SD_Cas and SD_PE. This implies that the interfacial area in SD_FO is smaller than that in SD_Cas and SD_PE, which limits interactions between the oil phase and prooxidant species such as transition metal ions. Since lipid oxidation is often catalyzed by metal ions (e.g., Fe^2^^+^ or Cu^2^^+^), a larger interfacial area increases the likelihood of interactions between these catalytic ions and the oil phase at the oil–water interface. Consequently, emulsions with smaller droplet sizes, and thus larger interfacial areas, may exhibit greater susceptibility to oxidation if not adequately stabilized. These results are in agreement with those obtained in previous studies on dressings fortified with fish oil microcapsules [17]. Interestingly, although the droplet size of SD_PE was smaller than that of SD_Cas, it exhibited superior oxidative stability. This suggests that factors beyond droplet size, such as interfacial properties, may also play a significant role in governing lipid oxidation in dressings fortified with fish oil emulsions.

The differences in volatile compound profiles between SD_PE and SD_Cas may be attributed to the properties of the oil–water interfaces. SD_PE exhibited lower concentrations of volatile oxidation compounds than SD_Cas, possibly due to the stabilizing effect of Pickering emulsions formed with the zein–caseinate Janus particles. The formation of these volatile compounds is strongly influenced by prooxidants, particularly transition metal ions [12]. Previous research has suggested that Pickering emulsions stabilized with zein‐caseinate Janus particles may limit the formation of volatile compounds by restricting prooxidants' access to the oil phase. In addition, while PV of SD_Cas and SD_PE were still below a critical threshold and tocopherol levels remained stable, the differences in the content of volatile compounds indicate that other factors could contribute to slowing down the formation of volatile compounds in SD_PE. These results suggest that the enhanced oxidative stability of SD_PE may be related to the structural advantages of the Pickering emulsion system, in which zein provides robust interfacial stabilization, and caseinate contributes antioxidant functionality. Together, these components form a dense interfacial layer with antioxidant activity that protects the oil phase from oxidation. This aligns with previous findings showing superior oxidative protection in Pickering emulsions stabilized with zein‐caseinate Janus particles compared to conventional emulsions stabilized with sodium caseinate [18].

### 3.3 Sensory Attributes of Salad Dressings

The results of the sensory evaluations are presented in Figure 5. Overall, the fish oil‐enriched salad dressings showed acceptable sensory properties as no significant sensory deterioration was observed during the storage. All samples were characterized by noticeable sour and vinegar‐like aromas. Notably, SD_PE received the lowest scores for glue‐like and green/hay odors, both of which are commonly associated with the early stages of rancidity. At week 4, the score for green/hay odors for SD_PE was significantly lower than those of SD_Cas and SD_FO, and there was a tendency for the glue‐like odor to be lower for SD_PE. Interestingly, despite these lower odor scores, the concentrations of several quantified volatile compounds in SD_PE were not consistently the lowest among the samples. This suggests that either (i) SD_PE may modulate the release or sensory perception of volatile compounds, or (ii) unpleasant odors may result from the combined effect of multiple volatile compounds rather than the presence of a single dominant compound [11]. Sensory evaluations of appearance and texture revealed no significant differences among SD_PE, SD_Cas, and SD_FO. However, a tendency toward increased lump formation was observed in SD_PE, while other textural and visual attributes remained largely unchanged. These findings indicate that the incorporation of fish oil emulsions had minimal effects on the appearance and texture of the salad dressings during storage.

**FIGURE 5:**
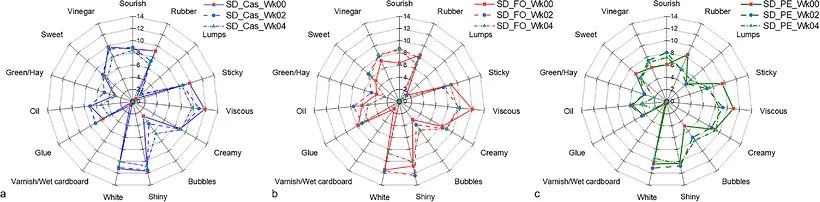
Sensory attributes of salad dressings SD_Cas (a), SD_FO (b), and SD_PE (c) at weeks 0, 2, and 4. Evaluated attributes included appearance (white, shiny, bubbles, and lumps), odor (sour, vinegar, sweet, green/hay, oil, glue, and varnish/wet cardboard), and texture (evaluated using a spoon; creamy, viscous, sticky, and rubbery).

## 4 Concluding Remarks

In general, this study demonstrated that the method of incorporating fish oil into salad dressings only slightly affected their physical and oxidative stability and sensory properties. All formulations showed high physical stability, exhibited slow lipid oxidation, and maintained acceptable sensory properties of the salad dressings throughout a 4‐week storage period. However, upon assessing the details, different characteristics emerged based on fortification approaches. No visible signs of physical instability were observed, while the increase in D[4,3] values during storage suggests potential droplet coalescence in dressings. In addition, a shoulder at 1.0 µm and stable D[3,2] values observed only in SD_PE suggest the presence of a stable population of small droplets associated with the Pickering emulsion structure. In terms of oxidative stability, only a slight increase in peroxide value was observed across all samples, while α‐tocopherol levels remained largely unchanged, suggesting slow lipid oxidation of the dressings. Among the formulations, SD_PE exhibited the slowest formation of lipid hydroperoxides and lower concentrations of key volatile compounds than SD_Cas, such as 1‐penten‐3‐ol. This suggests enhanced oxidative protection, which may be related to the unique advantages of Pickering emulsions stabilized with zein–caseinate Janus particles, where zein provides robust interfacial stabilization, and caseinate contributes antioxidant functionality. Sensory evaluations revealed no significant deterioration across all samples, with SD_PE receiving the lowest scores for off‐odors typically associated with rancidity, such as glue and green/hay notes. For future research, formulations with higher fish oil content or extended storage durations should be investigated to more clearly elucidate the effects of Pickering emulsions on the physicochemical characteristics, fatty acid compositions, and sensory properties of fortified food products. Furthermore, sensory evaluation of taste could not be conducted in this study because the samples were not produced to food‐grade standards. However, further evaluation of the taste could provide useful insights into the effects of incorporating fish oil into salad dressings via different approaches. Although SD_FO maintained the lowest concentration of volatile compounds, findings from this study highlight the potential of Pickering emulsions as an effective strategy for maintaining physical stability, while enhancing oxidative and sensory stability of omega‐3 delivery systems.

## Funding

This project has received funding from the European Union's Horizon 2020 research and innovation program under the Marie Skłodowska–Curie grant agreement No 956248.

## Conflicts of Interest

The authors declare no conflicts of interest.

## Supporting information




**Supporting File**: mnfr70612‐sup‐0001‐SuppMat.docx.

## Data Availability

The data that support the findings of this study are available from the corresponding author upon reasonable request.
