# Supplementary material for: Physicochemical Stability and Sensory Properties of Salad Dressing Formulated With Fish Oil‐in‐Water Pickering Emulsions
Source: Mol Nutr Food Res. 2026 Oct 12;70(19):e70612. doi: 10.1002/mnfr.70612 (PMC13655645; doi:10.1002/mnfr.70612)
Supplement: Supplementary file 1 [file MNFR-70-e70612-s001.docx]

**Physicochemical stability and sensory properties of salad dressing formulated with fish oil-in-water Pickering emulsions**

Thang Tran^1^, Grethe Hyldig^1^, Betül Yesiltas^1^, Ana C. Mendes^1^, Ioannis S. Chronakis^1^, Charlotte Jacobsen^1^^*^

^1^National Food Institute, Technical University of Denmark, Denmark

^*^Corresponding author

Figure S1. Content of tocopherols during storage experiments.

Figure S2. Formation of volatile compounds from salad dressings during storage experiment, (a) 2-ethylfuran, (b), 1-penten-3-one, (c) 2,4-heptadienal, and (d) Hexanal.
